# Sex differences in muscle morphology of the knee flexors and knee extensors

**DOI:** 10.1371/journal.pone.0190903

**Published:** 2018-01-23

**Authors:** Fearghal P. Behan, Thomas M. Maden-Wilkinson, Matt T. G. Pain, Jonathan P. Folland

**Affiliations:** 1 Arthritis Research UK Centre for Sport, Exercise and Osteoarthritis, Loughborough University, Leicestershire, United Kingdom; 2 School of Sport, Exercise, and Health Sciences, Loughborough University, Leicestershire, United Kingdom; 3 Faculty of Health and Wellbeing, Collegiate Campus, Sheffield Hallam University, Sheffield, United Kingdom; University of Memphis, UNITED STATES

## Abstract

**Introduction:**

Females experience higher risk of anterior cruciate ligament (ACL) injuries; males experience higher risk of hamstring strain injuries. Differences in injury may be partially due to sex differences in knee flexor (KF) to knee extensor (KE) muscle size ratio and the proportional size of constituent muscles.

**Purpose:**

To compare the absolute and proportional size, and mass distribution, of individual KE and KF muscles, as well as overall size and balance (size ratio) of these muscle groups between the sexes.

**Methods:**

T1-weighted axial plane MR images (1.5T) of healthy untrained young males and females (32 vs 34) were acquired to determine thigh muscle anatomical cross-sectional area (ACSA). Maximal ACSA (ACSAmax) of constituent muscles, summated for KF and KE muscle groups, and the KF:KE ratio were calculated.

**Results:**

Females had 25.3% smaller KE ACSAmax (70.9±12.1 vs 93.6±10.3 cm^2^; P<0.001) and 29.6% smaller KF ACSAmax than males (38.8±7.3cm^2^ vs 55.1±7.3cm^2^; P<0.001). Consequently, females had lower KF:KE ACSA ratio (P = 0.031). There were sex differences in the proportional size of 2/4 KE and 5/6 KF. In females, vastus lateralis (VL), biceps femoris long-head (BFlh) and semimembranosus (SM) were a greater proportion and sartorius (SA), gracilis (GR) and biceps femoris short-head (BFsh) a smaller proportion of their respective muscle groups compared to males (All P<0.05).

**Conclusion:**

Sex differences in KF:KE ACSAmax ratio may contribute to increased risk of ACL injury in females. Sex discrepancies in absolute and proportional size of SA, GR, VL and BFlh may contribute further anatomical explanations for sex differences in injury incidence.

## Introduction

Sex differences in the risk of specific sports injuries have been widely demonstrated; females have a higher risk of anterior cruciate knee ligament (ACL) injury [1,2] whilst males have a higher risk of hamstring strain injury (HSI) [3,4]. Females have also been demonstrated to suffer from a higher risk of knee osteoarthritis (OA) [5,6]. Various anatomical [7,8] and biomechanical [9,10] differences between sexes have been documented and suggested to contribute to the disparities in injury/disease risk. However, possible differences in the morphology of knee joint muscles between the sexes has received relatively little attention and could play a significant role in the observed sex differences in injury/disease risk.

The sex discrepancy in ACL injury incidence is particularly stark with females demonstrating a 3–5 times higher risk of sustaining an ACL injury than males when participating in agility sports (e.g. basketball, soccer, volleyball [11]). A commonly cited contributor to this discrepancy is the observation that females have a lower hamstrings to quadriceps (H/Q) strength ratio than males [12–15] which is thought to reflect reduced capacity for muscular stabilisation of the knee [16,17]. Quadriceps contraction elicits anterior tibial translation [15], particularly when the knee is close to full extension, which can load and ultimately rupture the ACL [18]. Contrastingly, hamstring contraction counteracts anterior tibial shear and may protect the ACL by improving dynamic joint stability [15,16].

The greater H/Q strength ratio of males vs females does not appear to be accounted for by differences in neural drive to the quadriceps and hamstrings muscles [14], and therefore our previous work hypothesised that females may simply have a disproportionately smaller hamstring muscle [14]. Whilst it is well known that females have smaller muscles than males, and that this is the case for both the quadriceps [19] and hamstrings [7], the relative size of these muscles has not been examined. A disproportionately small hamstrings muscle in females (i.e. low H/Q size ratio) might represent a fundamental anatomical difference between the sexes, that would be expected to result in a low H/Q strength ratio [20] and may predispose to knee joint injury.

Sex discrepancies in hamstring strain injuries (HSI) have been demonstrated with males experiencing a higher incidence than females (22.4±3.4 vs 11.5±2.6 injuries per 1000 athletes respectively [3]). HSI prevalence varies between the individual hamstring muscles, with a much higher prevalence in the biceps femoris long head (BFlh) compared to the semimembranosus or semitendinosus [21,22]. Males may have a relatively small BFlh in comparison to the whole hamstrings group, which could expose this muscle to greater load and injury risk. However, hamstrings muscle morphology between the sexes has not previously been compared.

Quantification of thigh muscle size may also allow for evaluation of the contribution of accessory knee flexor muscles other than just the hamstrings, such as the sartorius and gracilis. Previous research has commonly used the terminology: ‘hamstrings to quadriceps ratio’ when describing knee flexors (KF) and knee extensors (KE) torque ratios [14–16], which is somewhat simplistic considering that the sartorius, gracilis, popliteus and gastrocnemius are also agonist muscles for knee flexion. Therefore, ‘knee flexion to knee extension ratio’ may be a more appropriate and accurate term. The effects of these accessory KF have rarely been discussed in the literature despite the significant role of the sartorius and gracilis in controlling knee valgus/varus loading [23–25], which may contribute to acute injury risk and sex differences in knee joint loading. Moreover, any sex differences in knee joint muscle morphology would be expected to influence joint loading and stability and thus the joint degeneration that typically occurs with ageing and can lead to knee OA, with evidence for a greater incidence, particularly of aggressive OA, in females [5].

The aim of this study was to investigate knee joint muscle morphology, specifically absolute and proportional size, and mass distribution, of individual knee extensor and flexor muscles, as well as overall size and balance (size ratio) of these muscle groups, between sexes. It was hypothesised that males would have a significantly larger KF:KE muscle size ratio and females would have a larger biceps femoris long head as a proportion of the KF muscle group than males.

## Methods

### Participants

Sixty-six healthy, young, participants with a low-moderate level of physical activity (34 females, 32 males) provided written informed consent prior to their participation in this study, which was approved by the Loughborough University Ethical Advisory Committee. Participants had a BMI of ≤26 kg.m^-2^, no history of traumatic lower limb injury (ACL rupture, fracture etc.) or current musculoskeletal condition, and no experience with systematic physical training. Body mass and height were measured using a calibrated scale and stadiometer (Seca, Hamburg, Germany). Participants’ physical activity level was assessed using the International Physical Activity Questionnaire (iPAQ) short format [www.ipaq.ki.se/downloads.htm] [26]. Participants were advised not to undertake any unaccustomed/strenuous physical activity for 36 hours prior to their laboratory visit and to arrive in a relaxed state, having eaten and drunk normally, and to sit quietly for 15 minutes beforehand.

### Magnetic resonance imaging (MRI)

A 1.5 T MRI scanner (Signa HDxt, GE, CT, USA) was used to scan the dominant leg in the supine position with the hip and knee joints extended. T-1 weighted axial plane images were acquired from the anterior superior iliac spine to the knee joint space in two overlapping blocks and oil filled capsules were placed on the lateral side of the participants’ thigh to help with block alignment during analysis. The following imaging parameters were used: imaging matrix: 512 x 512 pixels, field of view: 260 mm x 260 mm, in plane spatial resolution: 0.508 mm x 0.508 mm, slice thickness: 5 mm, inter-slice gap: 0 mm.

**Fig 1:**
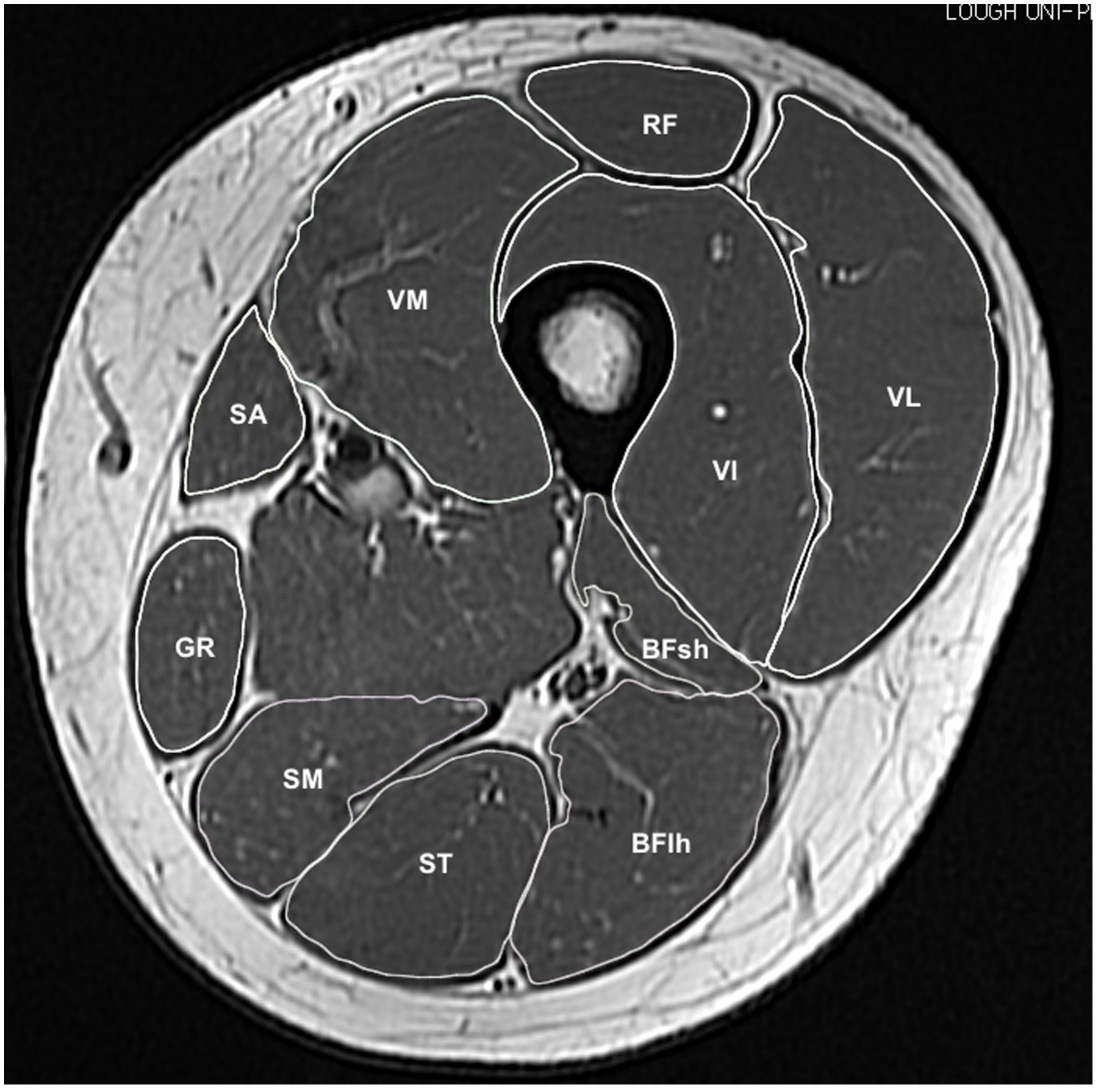
Example of MRI slice mid-thigh with the knee extensors and flexors manually segmented.

Image segmentation was performed manually with Osirix software (version 4.0, Pixmeo, Geneva, Switzerland). The knee extensor muscles: vastus medialis (VM), vastus lateralis (VL), vastus intermedius (VI), rectus femoris (RF) and the following knee flexor muscles: hamstrings (biceps femoris long head (BFlh), biceps femoris short head (BFsh), semitendinosus (ST), semimembranosus (SM)), sartorius (SA) and gracilis (GR) muscles were manually outlined in every third image starting from the most proximal image where the muscle first appeared (see Fig 1). Differentiation between VI and VL utilised the methods of Barnouin et al. [27]. One investigator conducted all the manual segmentation of KE, whilst a second investigator analysed KF. The maximal anatomical cross-sectional area (ACSA) of each muscle was defined as ACSAmax and summated KF and KE ACSAmax were calculated. This study used ACSA as the measure of muscle size rather than PCSA or muscle volume, due to concerns about: accurately determining physiological CSA [28], particularly in the 10 muscles assessed in this study; and that muscle volume may be confounded by the greater height and femur length of males. The size of each individual muscle’s ACSAmax was also expressed as a proportion (%) of the muscle group (KF/KE) ACSAmax. For example, for RF as a proportion of the KE the equation was as follows:

$$\frac{RFACSAmax}{\Sigma RFACSAmax+VMACSAmax+VIACSAmax+VLACSAmax}\times 100$$

The KF:KE ACSAmax ratio was calculated from these summations. ACSAmax was the criterion measure of muscle size in this study as it is strongly related to muscle strength [29], and not confounded by differences in muscle length as is the case for muscle volume. Nonetheless to compare muscle morphology along the length of femur muscle mass distribution of both sexes was calculated by expressing all ACSA values as a fraction of ACSAmax. Femur length (F_L_) was measured by analysing the distance between the most proximal (femoral head) and distal slices (lateral femoral condyle) in which the femur was apparent. The position of all the ACSA measurements along a muscle (i.e. from every slice) were expressed relative to F_L_ and ACSA values interpolated every 5%F_L_ using Matlab (Mathworks Inc., MA, USA). The intrarater reliability for ACSA calculated from the repeated analysis of six MRI scans was 0.4%.

### Statistical analysis

Data are presented as mean±SD. Sex differences in the KF:KE ACSAmax ratio were analysed using independent samples t-tests. Sex differences in ACSAmax of each muscle, sex differences in proportional size of each constituent muscle (relative to the whole muscle group e.g. VL ACSAmax as %KE ACSAmax) and sex differences in muscle mass distribution were analysed using two-way ANOVAs (Sex × muscle; Sex × muscle; Sex × F_L_, respectively); significant main effects were further examined with post-hoc t-tests with Holm-Bonferroni correction. Statistical significance was defined as P<0.05. Effect size was measured using Cohen’s D. All statistical procedures were performed with IBM SPSS Statistics for Windows (Version 22.0, NY, USA, IBM Corp.).

## Results

### Participant characteristics

Males were taller (1.78±0.07m vs. 1.68±0.06m, P<0.001) and heavier (71.8±7.2 vs. 62.9±7.2kg, P<0.001), but both groups were of similar age (males, 20.6±2.5 vs. females, 20.9±1.7yr). Females were more physically active than males (2,503±1,335 vs. 1,826±936 MET-mins week^−1^, P = 0.033) but both were categorised as moderately physically active [26].

### Sex differences in ACSA between KF and KE

**Fig 2:**
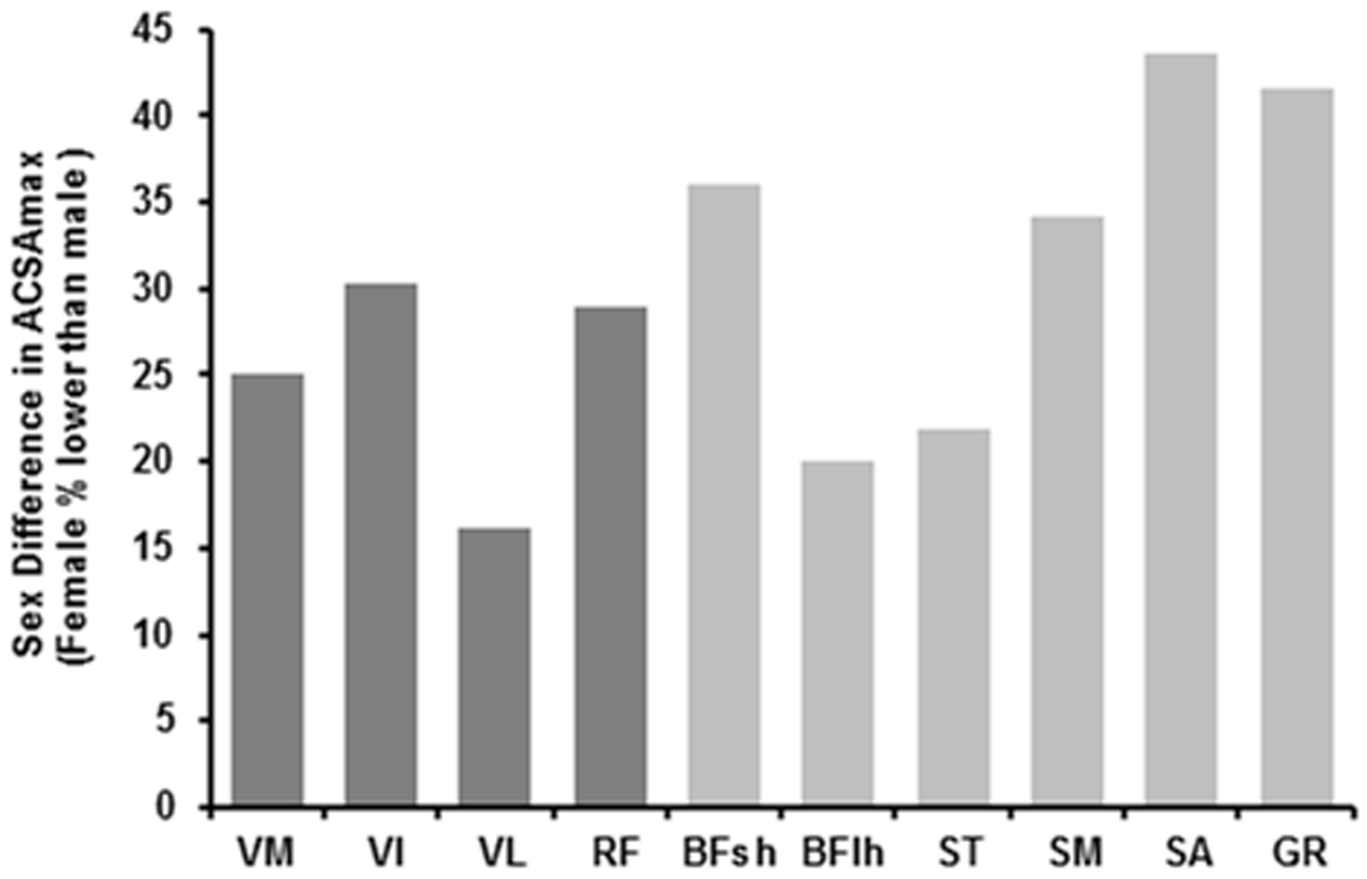
The magnitude of the sex difference for individual KE (dark grey) and KF (light grey) muscles. Data are presented as mean female ACSAmax (n = 34) as the percentage less than mean male ACSAmax (n = 32). The sex difference ranged from 16.1% for the VL to 43.6% for the SA.

**Fig 3:**
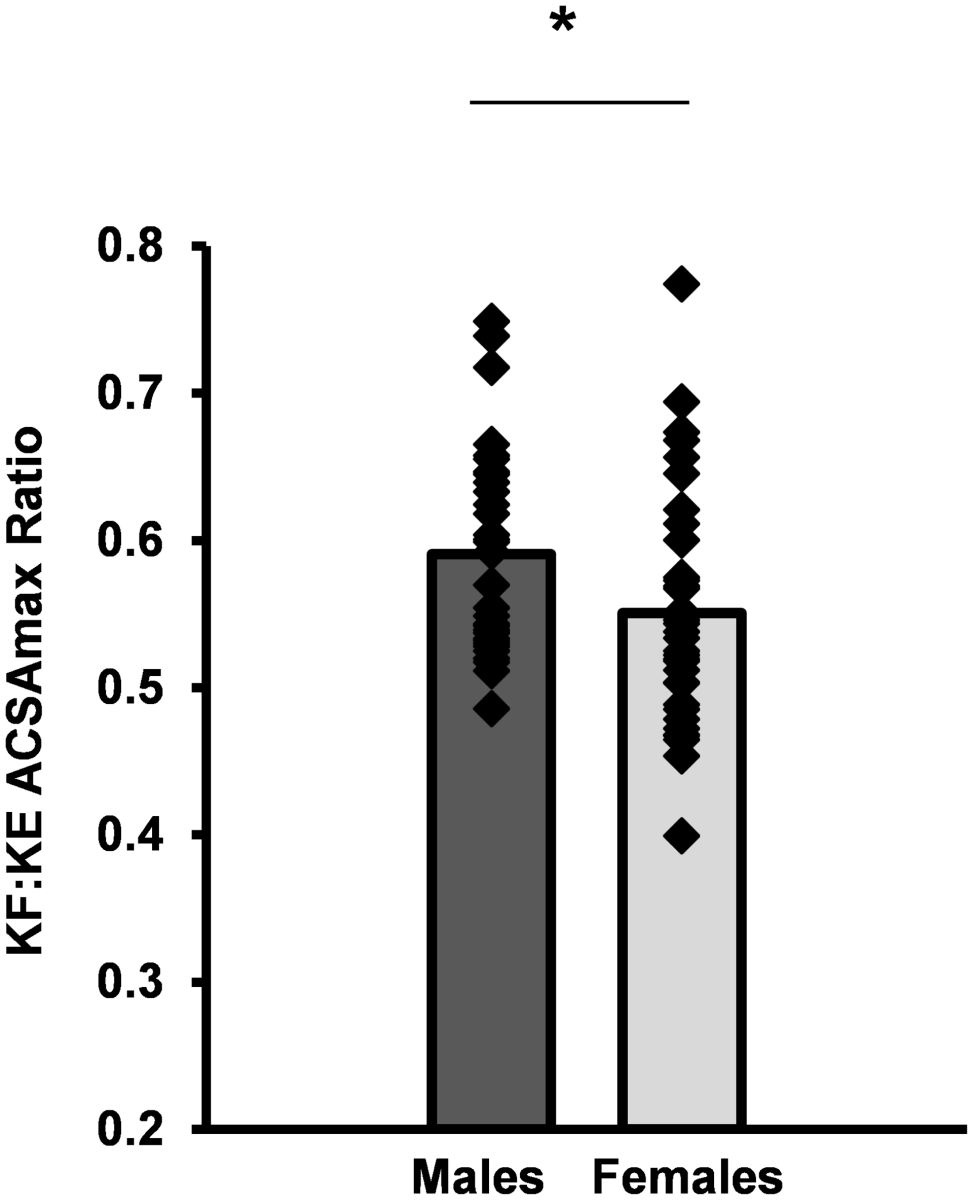
Knee flexors (KF) to knee extensor (KE) ACSAmax ratio for individual participants (diamonds) and mean for males (n = 32, dark grey bar) and females (n = 34 light grey bar). * P<0.05.

Females had smaller ACSAmax values for all individual KF and KE muscles than males (Table 1, P<0.001, S1 and S2 Files). However, the difference in ACSAmax for females compared to males ranged from -16.1% (VL) to -43.6% (SA), such that the sexual dimorphism was 2.7-fold greater for the SA than the VL (Fig 2). Females had a 25.3% smaller KE ACSAmax than males (70.9±12.1cm^2^ vs 93.6±10.3cm^2^) and 29.6% smaller KF ACSAmax than males (38.8±7.3cm^2^ vs 55.1±7.3cm^2^). Consequently, females had a lower KF:KE ACSAmax ratio (0.55±0.08 vs 0.59±0.07; P = 0.031; Table 1, Fig 3).

**Table 1:** Sex differences in ACSAmax of individual muscles and whole KF and KE muscle groups as well as the KF:KE ratio. Data presented as mean ± SD (range).

	Male (n = 32)	Female (n = 34)	P Value	Effect Size
KE (cm^2^)				
VM	24.3 ± 3.2 (19.2–34.9)	18.2 ± 3.5 (12.6–26.4)	<0.001	1.80
VI	26.2 ± 3.8 (19.2–34.1)	18.3 ± 3.6 (13.4–29.0)	<0.001	2.17
VL	29.2 ± 3.9 (22.5–38.3)	24.5 ± 5.2 (18.2–37.4)	<0.001	1.03
RF	13.9 ± 2.2 (9.1–18.5)	9.9 ± 2.0 (6.6–15.3)	<0.001	1.95
Total	93.6 ± 10.3 (75.0–118.1)	70.9 ± 12.1 (57.4–106.2)	<0.001	2.03
KF (cm^2^)				
BFsh	8.1 ± 3.1 (5.7–11.4)	5.2 ± 1.2 (3.5–8.7)	<0.001	2.16
BFlh	12.9 ± 2.2 (9.0–18.1)	10.3 ± 2.1 (7.6–15.6)	<0.001	1.21
SM	13.2 ± 2.8 (9.2–19.6)	10.3 ± 2.1 (6.2–14.9)	<0.001	1.18
ST	11.5 ± 2.5 (6.3–17.1)	7.6 ± 2.0 (4.4–11.8)	<0.001	1.77
SA	4.1 ± 0.6 (3.0–5.4)	2.3 ± 0.5 (1.3–3.4)	<0.001	3.17
GR	5.2 ± 0.9 (3.6–7.8)	3.1 ± 0.8 (1.9–5.3)	<0.001	2.45
Total	55.1 ± 7.3 (40.5–71.4)	38.8 ± 7.3 (28.4–56.7)	<0.001	2.25
KF:KE Ratio	0.59 ± 0.07 (0.49–0.75)	0.55 ± 0.08 (0.40–0.77)	0.031	0.59

### Sex differences in proportional size of constituent muscles

**Fig 4:**
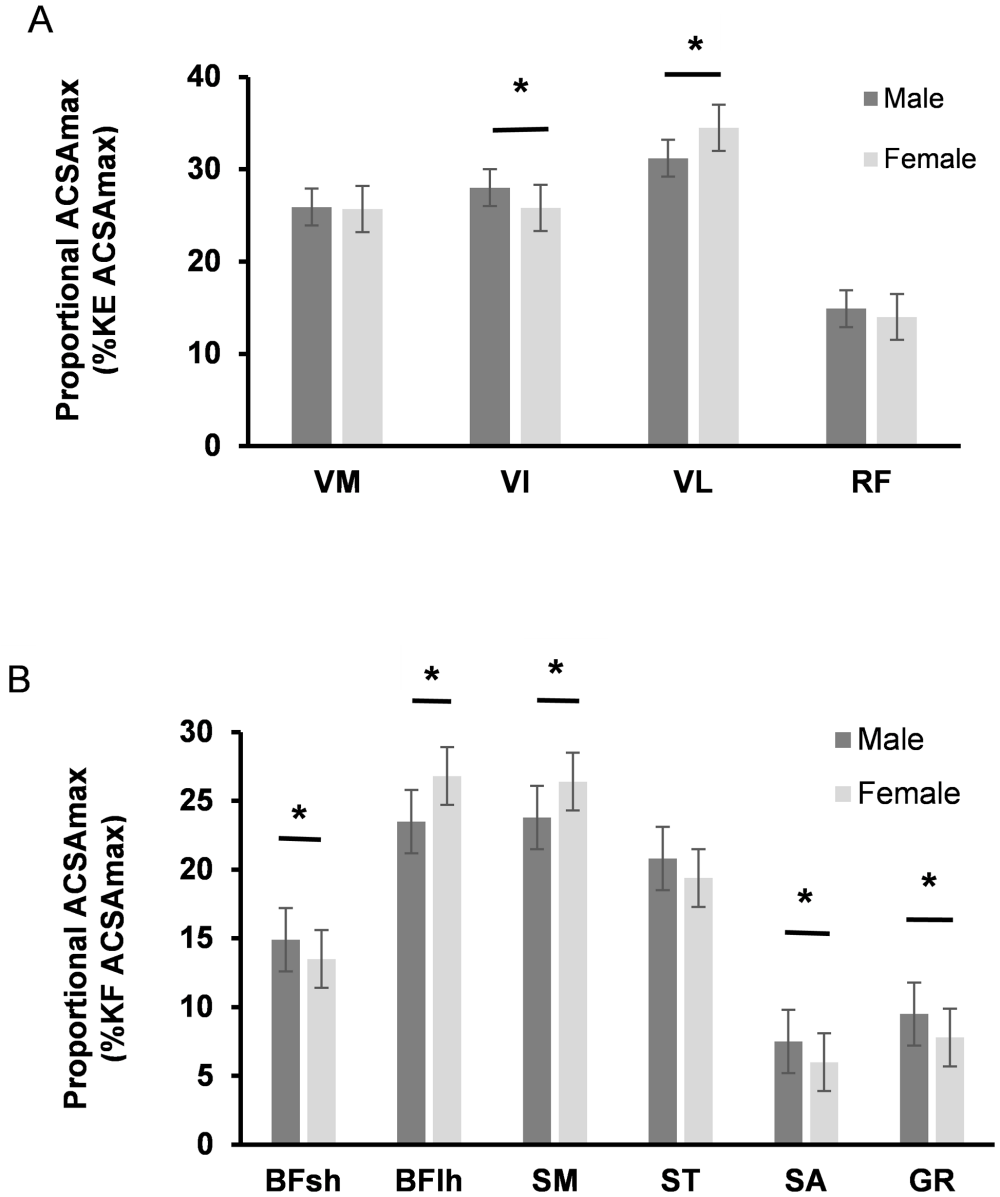
Proportional size of individual muscles relative to whole muscle group size (ACSAmax %knee extensor (A) %knee flexor (B) ACSAmax). Data are mean±SD of males (n = 32) and females (n = 34). * P<0.05.

There were sex differences in the proportional size of 2/4 constituent KE and 5/6 constituent KF muscles. In females, VL (34.5±3.1 vs 31.2±2.3%, P<0.001, Cohen’s D (d) 1.21) was a greater proportion and VI (25.8±2.4 vs 28.0±2.2%, P<0.001, d 0.96) a smaller proportion of the KE than in males (Fig 4A). Additionally, in females BFlh (26.8±2.8 vs 23.5±2.6%, P<0.001, d 1.23) and SM (26.4±3.2 vs 23.8±3.3%, P<0.001, d 0.80) were a greater proportion and SA (6.0±0.9 vs 7.5±1.0%, P<0.001, d 1.60), GR (7.8±1.2 vs 9.5±1.5%, P<0.001, d 1.28) and BFsh (13.5±1.7 vs 14.9±2.3%, P<0.001, d 0.68) a smaller proportion of the KF than in males (Fig 4B).

### Sex differences in muscle mass distribution

**Fig 5:**
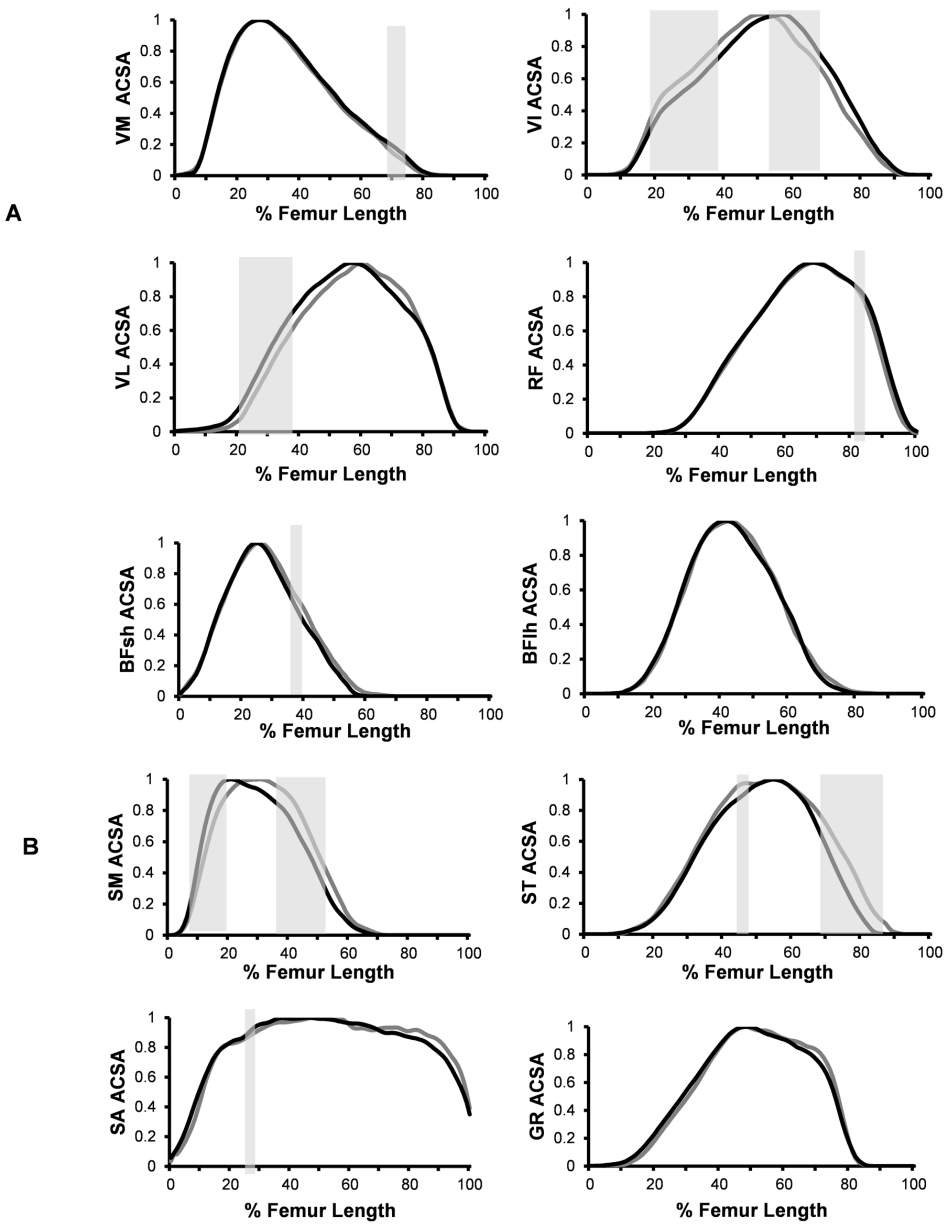
Muscle mass distribution of males (n = 32 black line) and females (n = 34 dark grey line). Mean normalised ACSA (%ACSAmax) of the individual knee extensors (A) and knee flexors (B) along the length of the femur (distal: 0% to proximal:100%). Areas of significant sex differences are shown by light grey shading (P<0.05).

The ACSA values of each muscle for both sexes, interpolated every 5%F_L_ and normalised to ACSAmax, showed sex differences in muscle mass distribution for all 4 KE and 4/6 KF, the exceptions being BFlh and GR (Fig 5). For the KE, the differences in mass distribution were subtle for VM and RF, but were over greater regions of the VL and VI. For KF, sex differences in muscle mass distribution were subtle for BFlh and SA while differences were found over greater regions in SM and ST.

## Discussion

This study investigated sex differences in muscle morphology (absolute and proportional size, and mass distribution) of individual knee extensor and flexor muscles, as well as overall size and size ratio of these muscle groups. There were a range of sex differences in muscle morphology that may predispose females to greater risk of ACL injury, primarily, as previously hypothesised [14], a smaller KF:KE size ratio, but also a proportionately small SA and GR and a proportionately large VL. Our finding of a fundamental difference in the balance of muscle morphology across the knee provides a likely explanation for the lower KF:KE torque ratio of females [12–15], would be expected to reduce the functional stability of the knee joint and thus may be a key factor in the greater incidence of ACL injuries in females. The clear differences in the proportional size of the constituent KE (larger VL in females) and KF (smaller GR and SA, but larger BFlh in females) further highlighted the extent of the sexual dimorphism in muscle size within the thigh musculature. This data also confirmed our second hypothesis that females have a larger BFlh as a proportion of the KF than males, which may contribute to the higher risk of HSI in males. In addition, the different proportions of the constituent KE and KF muscles may result in long-term differences in knee stability and loading across the knee joint and thus contribute to the sex disparity in OA [30].

It is well known that females have smaller muscles than males [8,31,32], this was the case for all KF and KE in this study. This consistent sex difference in muscle size is widely attributed to lower levels of androgenic hormones, and particularly testosterone [33], in females. Females had smaller KE (25%) and KF (30%) than males, and the greater disparity in KF resulted in the lower female KF:KE ACSAmax ratio. Our previous work found the lower KF:KE strength ratio of females was not accounted for by differences in neural drive and led to the hypothesis that females may simply have disproportionately smaller KF [14]. The current results confirm this hypothesis with females having a lower KF:KE size ratio (females 0.55 vs males 0.59) equivalent to a 7% smaller KF group in relation to the KE. This size ratio difference is consistent with the common observation of lower KF:KE strength ratio in females [12–15] and similar in magnitude to our previous findings for strength ratio differences (50 vs 56%) [14]. Quadriceps contraction elicits anterior tibial translation, particularly when the knee is close to full extension, which can load and ultimately rupture the ACL [18]. Contrastingly, KF contraction counteracts anterior tibial shear and may protect the ACL by improving dynamic joint stability [34,35]. This disproportionately smaller KF muscle group relative to KE in females may reduce the ability to counteract the anterior tibial translation and may be a key factor in females’ greater incidence of ACL injuries. On an individual basis, there were a wide range of KF:KE size ratios within both sexes with some high values (>0.65) amongst both sexes. However, 6 females had a KF:KE size ratio of <0.50 compared to only 1 male; these individuals may be particularly at risk of ACL injury. Resistance training is known to increase muscular size and strength [36] therefore, these results may indicate the importance of targeted KF resistance training for injury prevention in females, especially those with a low KF:KE strength/size ratio.

Whilst all the KE and KF muscles were smaller in females the difference was highly variable with differences from 16% (VL) to 44% (SA) smaller. Consequently, the proportional size of the constituent muscles within the KE and KF also displayed a marked sex difference that would also be expected to contribute to the discrepancy in ACL injury incidence. Females had substantially smaller SA (44%) and GR (42%), subsequently these muscles were a smaller proportion of the KF. The large sex disparity between these KF muscles (SA, GR) contributed to the lower KF:KE ACSAmax ratio. Considering that the SA and GR are known to be important for controlling valgus knee forces [23–25,37], this sex discrepancy in SA and GR ACSAmax may also be an important factor in females’ greater ACL injury risk. The SA also acts as a hip external rotator, that likely reduces the hip internal rotation associated with ACL injury mechanisms [10]. Further investigations utilising musculoskeletal models with the ability to isolate the effects of increased torque production of these specific muscles are warranted.

The finding that females have a significantly larger VL as a proportion of the KE than males is noteworthy. The VL produces valgus moments at the knee between knee flexion angles of 20–50° [37] and in combination with BF, which was also found to be proportionately higher in females, this muscle has been demonstrated to increase ACL elongation [38]. The proportionately larger VL of females may provide a further anatomical explanation for increased female ACL injury risk.

The increased BFlh as a percentage of KF ACSA in females may have significant relevance regarding HSI. Males have been found to have a higher risk of HSI than females [3] and the most commonly injured hamstring muscle is the BFlh [21,22]. HSI typically occurs during late stage swing phase when BFlh is undergoing an eccentric contraction [39,40]. A proportionally larger BFlh within the KF would be expected to increase the contribution of this muscle to eccentric knee flexion strength [20], reducing the risk of eccentric overload in this muscle and thus contribute to the lower risk of female HSI. A greater risk of HSI has previously been linked to a low H/Q strength ratio [41], but our findings of a lower size ratio in females that are known to experience less HSIs, indicate that this ratio may not be important for explaining the sexual dimorphism in HSI.

Sex differences in muscle mass distribution were observed in most of the muscles examined, but were typically subtle. Although previous research compared sex differences in muscle mass distribution with a single value (muscle shape factor: mean ACSA as a fraction of ACSAmax [19]) this study compared mass distribution along the entire muscle length. However, the functional implications of these sex differences in muscle mass distribution are currently unknown. Future investigations should compare all aspects of muscle-tendon unit morphology of knee joint musculature between the sexes including architecture, ideally using 3-D diffusion tensor MRI that would facilitate calculation of physiological CSA, as well as tendon and aponeurosis morphology.

The current study has some limitations. Firstly, it did not include the full complement of KF with popliteus and gastrocnemius excluded. Future studies would benefit from inclusion of these muscles to evaluate all KF muscles to investigate any further sex differences in muscle morphology than the current findings. Secondly, despite all participants being recruited to have low-moderate level of physical activity, the iPAQ revealed a sex difference in physical activity (females>males). However, both sexes were both still categorised as moderately physically active [26], and given that none of the participants had any background of strength/power training it seems unlikely that this would explain the observed differences in muscle morphology and it seems probable that these differences are innate. Future studies should examine sex differences in knee joint muscle morphology for individuals participating in agility sports with a high incidence of ACL/HSI. Although every attempt was made to remove visible non- contractile tissue from the analysis, some non- contractile tissue may have been included in the analysis. Any non- contractile tissue included would have been similar for both sexes and expected to have minimal implications on the findings. Finally, as sex differences in muscle moment arms [42], muscle architecture [43] and the area occupied by different muscle fiber types [44] could influence muscular torque production, these factors should be included in future investigations alongside muscle size measurements.

In conclusion, there was a sex dimorphism in muscle size both between (i.e lower KF:KE ACSAmax ratio of females) and within knee joint muscle groups (disproportionately larger VL and BFlh, but smaller SA and GR in females). These findings would appear to contribute to females’ greater risk of ACL injuries potentially by 3 separate mechanisms (lower KF:KE muscle balance, disproportionately smaller SA/GR, disproportionately larger VL) and highlight the importance of KF development with resistance training for prevention of ACL injuries in females. The findings from this study may form a foundation for future intervention studies aimed at positively altering muscle size profiles of females to reduce their potential risk of ACL injuries and for males to reduce HSI.

## Supporting information

S1 File Male MRI data. (XLSX)

S2 File Female MRI data. (XLSX)
